# Analysis of influencing factors of atmospheric pollution on pulmonary tuberculosis incidence in China from 2014 to 2020

**DOI:** 10.3389/fpubh.2026.1951817

**Published:** 2026-10-14

**Authors:** Haibo Shi, Xinyu Guo, Yangzong Ciren

**Affiliations:** Xizang University, Lhasa, Xizang, China

**Keywords:** air pollution, China, geographically and temporally weighted regression, pulmonary tuberculosis, spatial autocorrelation

## Abstract

**Objective:**

To investigate the spatiotemporal heterogeneity in the associations between ambient air pollutants (PM_2.5,_ SO_2_, NO_2_, CO, O_3_) and pulmonary tuberculosis (TB) incidence in China from 2014 to 2020, and to provide evidence for region-specific prevention and control strategies.

**Methods:**

Based on panel data from 31 provincial-level regions, spatial autocorrelation analysis, spatiotemporal scanning, and a Geographically and Temporally Weighted Regression (GTWR) model were employed. An exposure-window robustness analysis using 1- to 5-year moving averages was further conducted.

**Results:**

The overall TB incidence declined, yet high-burden clustering remained significant in Xinjiang, Xizang, and Qinghai (RR = 3.56). The GTWR model showed good fit (*R*^2^ = 0.889). PM_2.5_ coefficients exhibited an “east-negative, west-positive” gradient; SO_2_, NO_2_, and CO showed synergistic positive associations in Northeast China; O_3_ was negatively associated in most provinces and attenuated as the exposure window lengthened, suggesting predominantly short-term associations; and GDP coefficients reversed from positive in the east to negative in the west. The moving-average analysis confirmed that these spatial patterns were temporally robust.

**Conclusion:**

The associations between air pollution and pulmonary TB exhibit marked spatiotemporal non-stationarity, supporting region-specific prevention and control strategies prioritizing high-burden western regions. As an ecological study, this investigation identifies associations rather than causal relationships.

## 1 Introduction

Environmental air pollution remains a significant public health threat globally, with approximately 99% of the population exposed to pollutants daily (1). As a major developing nation undergoing rapid industrialization and urbanization, China continues to face severe air pollution challenges. An analysis of air quality monitoring data from 336 cities across the country—covering the years 2014 to 2019—indicates that concentrations of key pollutants, including PM₂.₅, PM₁₀, SO₂, NO₂, and CO, generally declined during this period. However, pollutant levels in northern, northwestern, and central Henan regions remained notably elevated (2). Notably, the annual mean PM₂.₅ concentration in many of these regions still exceeded both the Chinese Ambient Air Quality Standard (GB 3095–2012; 35 μg/m^3^) (3) and the World Health Organization(WHO) Air Quality Guideline (5 μg/m^3^) (4), with NO₂ levels similarly surpassing the national (40 μg/m^3^) and WHO (10 μg/m^3^) annual limits. Exposure to air pollution is closely linked to the onset, exacerbation, and mortality of respiratory diseases such as chronic obstructive pulmonary disease, lung cancer, and asthma. Additionally, it can heighten the risk of respiratory infectious diseases, including pneumonia, bronchitis, and tuberculosis (5).

Tuberculosis is a chronic respiratory infectious disease caused by *Mycobacterium tuberculosis* and transmitted through respiratory droplets. Each year, over 2 million individuals succumb to tuberculosis globally (6). Although global strategies for tuberculosis control are evolving, developing nations continue to face a significant burden. In China, the reported incidence rate of pulmonary tuberculosis has declined from 73.3 per 100,000 in 2004 to 55.45 per 100,000 in 2018. Despite this decrease, the disease burden remains substantial, with persistent high-incidence clusters in the western, central, and southern regions (7, 8).

Environmental air pollution is a modifiable risk factor, and recent epidemiological studies have quantitatively explored its association with tuberculosis risk (9–11). Time-series analyses conducted in various Chinese cities have provided compelling evidence linking air pollution to tuberculosis incidence. For instance, research in Chengdu indicates that when concentrations of PM₁₀ (RR = 1.06), NO₂ (RR = 1.07), and SO₂ (RR = 1.07) surpass specific thresholds, the relative risk of new active pulmonary tuberculosis cases increases with rising pollutant levels (12). A multi-city study encompassing 67 districts and counties in China further revealed that elevated levels of SO₂, NO₂, PM₁₀, PM₂.₅, and CO are positively correlated with tuberculosis risk, with lag times ranging from 0 to 15 weeks (13).

National model analyses substantiate the role of particulate matter in health outcomes. Specifically, for every 10 μg/m^3^ increase in PM₂.₅, the risk of tuberculosis rises by 12%, while a similar increase in PM₁₀ correlates with a 7% increase in risk (14). A causal inference study utilizing long-term data from 1982 to 2019 also identified a significant driving effect of PM₂.₅ on tuberculosis incidence (15). Evidence at the urban level corroborates these findings: a long-term analysis in Lianyungang demonstrated that exposure to PM₂.₅ (RR = 1.12), PM₁₀ (RR = 1.11), SO₂ (RR = 1.37), and NO₂ (RR = 1.39) is associated with an elevated risk of active pulmonary tuberculosis (16), and time-series data from Nantong reported significant effects of PM₂.₅, PM₁₀, and NO₂ on tuberculosis risk (17).

However, O₃ has not demonstrated a significant association with tuberculosis incidence in multiple investigations (14, 17), and the combined effect of CO remains unclear (10). The reported effect size for the same pollutant differs considerably across regions. This heterogeneity may be attributed to factors including pollutant composition, climatic conditions, population immune status and social-economic status (11). Furthermore, the influence of air pollution on tuberculosis may differ by region and time period (7).

Experimental evidence substantiates the biological mechanism through which air pollutants increase the risk of tuberculosis. Pre-exposure to PM₂.₅ diminishes the capacity of peripheral blood mononuclear cells to regulate the growth of *Mycobacterium tuberculosis*, inhibits CD69 expression induced by *Mycobacterium tuberculosis*, reduces the production of critical cytokines such as IFN-γ and TNF-α, and concurrently upregulates the anti-inflammatory factor IL-10 (18). The accumulation of particulate matter in alveolar macrophages correlates with alterations in cytokine production induced by *Mycobacterium tuberculosis*, indicating that inhalation of urban air particulate matter may compromise the anti-tuberculosis immune response in both the lungs and the body as a whole (19). Respiratory epithelial cells are also directly affected by particulate matter exposure: PM₂.₅ and PM₁₀ can induce senescence in A549 cells while downregulating the expression of human β-defensin 2 and 3, thereby promoting the proliferation of *Mycobacterium tuberculosis* within these cells (20). Collectively, these experimental findings may explain a biological mechanism underlying the epidemiological link between air pollution and the onset of tuberculosis.

The current epidemiological evidence, however, is constrained by methodological limitations. Most studies rely on aggregated data from individual cities or regions and employ time-series or global regression frameworks, which typically assume that pollutant effects are constant over time and across space (11). Yet substantial disparities exist among Chinese provinces in socioeconomic development, healthcare resources, population mobility, climatic conditions, and pollutant composition. Moreover, the spatial distribution of tuberculosis incidence exhibits clear clustering (7, 8), suggesting that the constancy assumption may obscure critical local variations. In light of this, the present study employs a Geographically and Temporally Weighted Regression (GTWR) framework, using panel data from 31 provinces in China spanning 2014 to 2020. This approach aims to assess the spatiotemporal heterogeneous associations between exposure to PM₂.₅, SO₂, NO₂, CO, and O₃ and the incidence of pulmonary tuberculosis. The findings seek to provide an evidence-based foundation for regionally differentiated tuberculosis prevention and control strategies, as well as for decisions regarding air pollution management.

## 2 Materials and methods

### 2.1 Data sources

The tuberculosis data used in this study were obtained from the Tuberculosis Management Information System (TBIMS) of the China Public Health Science Data Center (https://www.phsciencedata.cn), a national public health data platform managed by the Chinese Center for Disease Control and Prevention (China CDC). The TBIMS aggregates case notifications from the China Information System for Disease Control and Prevention (CISDCP), the national legally notifiable infectious disease surveillance system. We extracted the reported incidence rate of pulmonary TB by province and year (per 100,000 population), calculated by dividing the number of newly reported confirmed cases in the current year by the average of the population at the beginning and end of the year for the corresponding province. Case classification was based on the national diagnostic criteria for pulmonary tuberculosis (WS 288–2017) to ensure consistency in case definitions across provinces and years.

Air pollutant data. Annual mean concentrations of six air pollutants—PM_2.5_, PM_10,_ SO_2_, NO_2_, CO, and O_3_—were obtained from the China Air Quality Online Monitoring and Analysis Platform (https://www.aqistudy.cn/historydata/). The original hourly concentration measurements were sourced from the National Real-time Urban Air Quality Monitoring Platform of the China National Environmental Monitoring Centre. For each city, daily mean concentrations were first calculated from hourly records, and annual city means were then computed. Provincial annual mean concentrations were obtained by averaging the annual city means across all monitored cities within each province. CO was measured in mg/m^3^, while the remaining five pollutants were measured in μg/m^3^.

Provincial gross domestic product (GDP, in billion yuan) and Passenger volume of public buses and trolleybuses (PVOPBAT, in million person-times) for 2014–2020 were obtained from the National Bureau of Statistics of China (https://www.stats.gov.cn) and corresponding provincial statistical yearbooks. GDP served as an indicator of economic development and health-service capacity, while PVOPBAT was treated as a proxy for population mobility and traffic-related exposure.

### 2.2 Research methods

#### 2.2.1 Spatiotemporal distribution of pulmonary TB incidence

This study employed an ecological study design to conduct a spatiotemporal analysis of the reported incidence rates of pulmonary tuberculosis in 31 provinces of China from 2014 to 2020. We calculated not only the crude incidence rates (per 100,000) by year, province, and age group, but also the age-standardized weighted incidence rates for each year. By introducing demographic weights, this study effectively corrected for age structure bias, thereby ensuring the objectivity and reliability of comparisons of spatiotemporal trends across years.

#### 2.2.2 Global spatial autocorrelation analysis

Global spatial autocorrelation analysis employed the Moran’s I index to measure the degree of spatial autocorrelation, with values ranging from −1 to 1. A Moran’s I greater than 0 indicates positive spatial autocorrelation (clustering of similar values); less than 0 indicates negative spatial autocorrelation (dispersion of similar values); and equal to 0 suggests a random spatial distribution.

Local spatial autocorrelation analysis used Local Indicators of Spatial Association (LISA) maps to detect clustering patterns in specific regions. LISA can identify four types of spatial clusters: high–high (clustering of high values), high–low (high values adjacent to low values), low–high (low values adjacent to high values), and low–low (clustering of low values). These analyses provide deeper insights into the local characteristics and clustering patterns of spatial data. The base map used in this study was sourced from the National Platform for Common Geospatial Information Services (review number: GS (2024) 0650). Due to the absence of reported incidence data, Hong Kong, Macao, and Taiwan were not included in the spatial analysis.

#### 2.2.3 Spatiotemporal scanning analysis

Spatiotemporal scanning statistics were performed using SaTScan v10.3.3 software to identify disease clustering characteristics. The main indicators included the number of observed cases, expected cases, relative risk (RR), log-likelihood ratio (LLR), and the corresponding *p*-value derived from 999 Monte Carlo simulations.

Observed cases: the actual number of pulmonary TB cases reported within a given scanning window (i.e., a candidate spatiotemporal cluster).

Expected cases: the number of cases expected within the same window if pulmonary TB incidence were randomly distributed across space and time, calculated based on the population at risk and the overall incidence rate of the study region.

Relative risk (RR): the ratio of pulmonary TB risk inside the scanning window to that in all remaining areas outside the window during the same time period. Specifically Equation 1,

RR=ObservedExpected insidetheclusterObservedExpected outsidethecluster (1)

An RR > 1 indicates an elevated risk inside the cluster relative to the outside reference population, with larger values suggesting stronger clustering. The reference group consists of all other provinces not included in that cluster.

Log-likelihood ratio (LLR): a statistical measure comparing the likelihood of the data under the alternative hypothesis (a cluster exists) to that under the null hypothesis (no cluster exists). It is calculated from the Poisson model as Equation 2:

$$\mathrm{LLR}=2\times [O_{\text{in}}\ln(\frac{O_{\text{in}}}{E_{in}}+O_{\text{out}}\ln(\frac{O_{\text{out}}}{E_{\text{out}}}))]\;\;\;\;\;$$ (2)

Where *O*_*in*._ and *E_in_* are the observed and expected cases inside the window, and *O*_*out*._ and *E_out_* are those outside. Higher LLR values indicate stronger evidence for the presence of a cluster.

*p*-value: obtained from 999 Monte Carlo replications to assess statistical significance. A cluster was considered significant when the *p*-value was less than 0.05.

The model was constructed based on a discrete Poisson probability distribution, with the temporal analysis precision set to year. For scanning window parameters, the upper limit of the spatial scanning radius was set to cover 50% of the at-risk population, and the upper limit of the temporal window was set to 50% of the study period, balancing detection sensitivity and specificity.

#### 2.2.4 Construction of the GTWR model

The GTWR model integrates spatial location and temporal sequence characteristics, enabling dynamic estimation of regression coefficients for each observation point and thereby reflecting local heterogeneity in variable relationships across time and space. By performing local regression in a three-dimensional spatiotemporal coordinate system, this method reveals spatial patterns that evolve over time and is thus widely applied in spatiotemporal heterogeneity analysis.

Before conducting multiple regression analysis, it is necessary to examine whether high correlations exist among independent variables, in order to avoid unstable parameter estimates associated with multicollinearity. Accordingly, we first performed pairwise Pearson correlation tests among all independent variables and obtained the corresponding correlation coefficients matrix. The variance inflation factor (VIF) was then used to further detect multicollinearity, with its calculation formula shown in Equation 3:

$$\mathrm{VIF}=1∕(1-r^{2})$$ (3)

In the formula, *R*^2^ represents the coefficient of determination between a given independent variable and the other independent variables.

The basic form of the GTWR model is shown in Equation 4:

$$y_{i}=\beta_{\begin{array}{c} 0 \end{array}}(u_{i},v_{i},t_{i})+\sum_{\begin{array}{c} k=1 \end{array}}^{m}\beta_{k}(u_{i},v_{i},t_{i})x_{\mathrm{ik}}+E_{i}\;$$ (4)

In the formula, yᵢ represents the dependent variable for the *i*-th sample; the intercept term and the regression coefficients for the k-th independent variable are functions of the longitude, latitude, and time coordinates of the i-th sample; x_ik_ is the k-th independent variable for the *i*-th sample; and εᵢ is the random error term. Through this model, dynamic changes in variable relationships across time and space can be captured, thereby revealing the complex spatiotemporal associations between TB incidence and factors such as air pollution (21).

To account for potential confounding by socio-economic and transportation-related factors, two additional covariates were incorporated into the GTWR model: annual regional gross domestic product (GDP, in 100 million yuan) and public passenger transport volume (PVOPBAT, in 10,000 person-times) for the 31 provinces from 2014 to 2020. GDP serves as a proxy for economic scale and living standards, which may influence both health-seeking behavior and healthcare resource allocation. PVOPBAT reflects the intensity of population mobility and traffic-related emissions, factors that could independently affect both air pollution levels and TB transmission dynamics. Data were sourced from the statistical yearbooks provided by the National Bureau of Statistics. These variables were included as covariates in the GTWR model to mitigate potential bias arising from regional economic development and transportation activities.

#### 2.2.5 Exposure-window sensitivity analysis using moving averages

To determine whether the spatiotemporal heterogeneity identified by GTWR reflected persistent geographic structures or transient annual fluctuations, a moving-average exposure-window analysis was conducted on the annual pollutant time series. For each pollutant (PM₂.₅, PM₁₀, SO₂, NO₂, CO, O₃), moving averages over 1, 2, 3, and 5 years (MA1, MA2, MA3, MA5) were computed based on the annual national mean concentrations, where MAk represents the average of the current and preceding k − 1 years. This procedure generated a series of exposure-window-specific GTWR estimates, enabling direct comparison of coefficient directions, magnitudes, and spatial patterns across time horizons. The analysis served to assess the temporal robustness of pollutant–TB associations and to characterize dose-window relationships. All GTWR modeling was performed in ArcGIS using the GTWR plug-in developed by Prof. Xin Huang (Wuhan University).

## 3 Results

### 3.1 Spatiotemporal distribution of pulmonary TB incidence

From 2014 to 2020, both the reported crude and age weighted incidence of pulmonary TB in China showed an overall decreasing trend (Figure 1). Significant differences in incidence were observed across provinces. Xizang and Xinjiang had markedly higher incidence rates than other regions. Xizang incidence reached 147.99 per 100,000 in 2014 and remained at 150.13 per 100,000 in 2020. In contrast, first-tier cities such as Beijing and Shanghai had relatively low incidence rates, with Beijing at 28.56 per 100,000 and Shanghai at 24.33 per 100,000 in 2020. Provinces such as Beijing and Guangdong showed continuous declines from 2014 to 2020, while Guizhou maintained relatively high levels from 2014 to 2019, declining to 96.54 per 100,000 in 2020. Yunnan incidence increased from 56.01 per 100,000 in 2014 to 60.07 per 100,000 in 2020, indicating a persistent TB burden (Table 1). Notably, Xinjiang incidence peaked at 304.94 per 100,000 in 2018.

**Figure 1:**
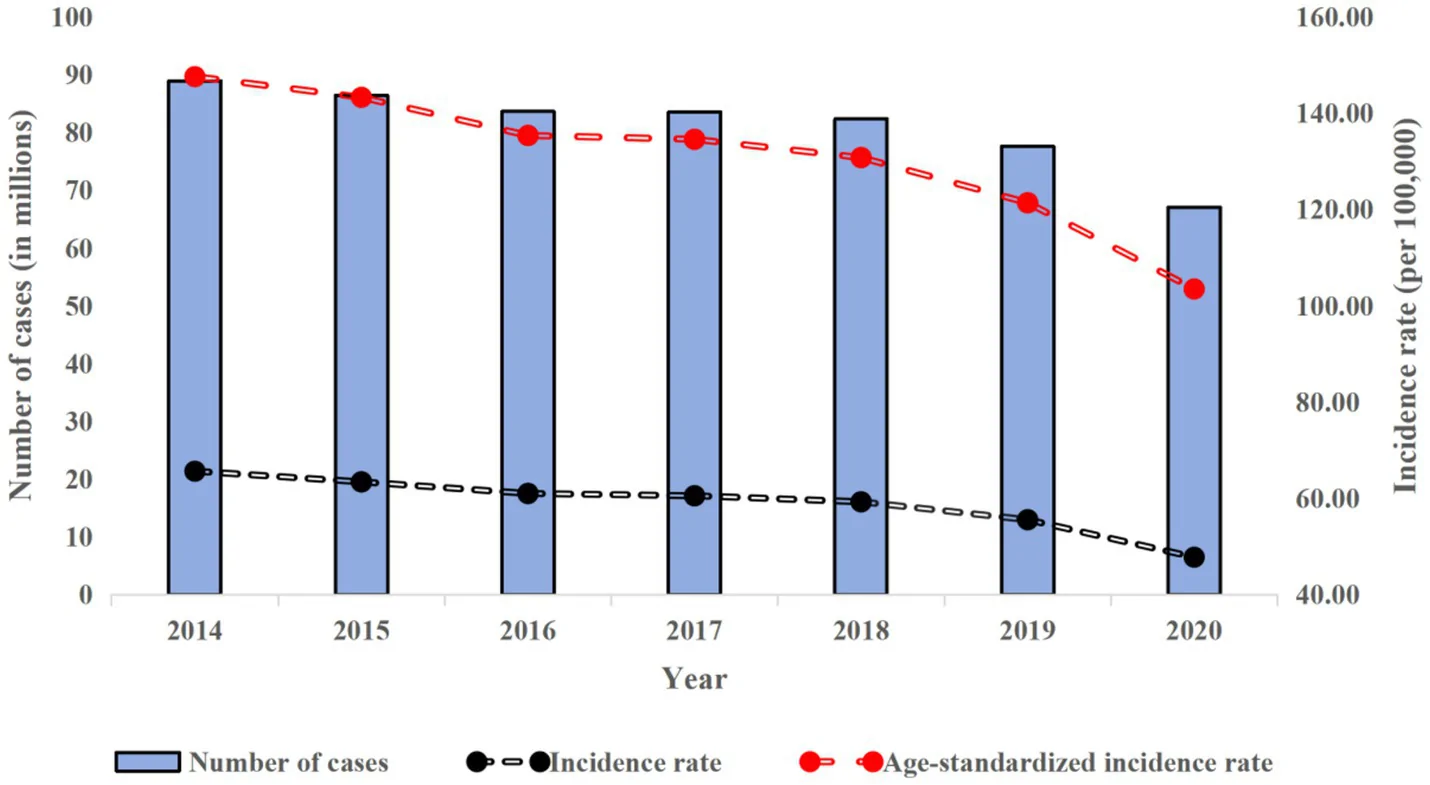
Reported national pulmonary TB cases in China, 2014–2020.

**Table 1:** Provincial pulmonary TB incidence in China, 2014–2020 (per 100,000 population).

Province	2014	2015	2016	2017	2018	2019	2020
Beijing	34.38	31.97	31.01	32.74	30.43	32.22	28.56
Tianjin	21.27	19.52	21.14	20.91	21.39	24.98	20.51
Hebei	48.15	46.11	45.34	41.94	39.31	39.24	32.93
Shanxi	46.33	42.32	38.65	37.22	35.03	33.88	29.74
Inner Mongolia	49.14	47.71	48.30	49.19	54.09	49.86	39.41
Liaoning	57.01	53.72	51.40	54.08	55.81	52.75	43.89
Jilin	60.66	56.45	49.76	50.30	47.43	39.98	31.76
Heilongjiang	89.43	86.73	80.16	72.73	65.23	59.57	40.83
Shanghai	28.74	27.56	27.28	26.55	26.14	26.72	24.33
Jiangsu	41.90	39.52	35.93	33.52	31.02	31.25	28.40
Zhejiang	54.05	50.80	48.78	47.54	45.26	45.79	41.92
Anhui	61.84	58.44	56.77	55.78	51.08	48.65	41.87
Fujian	47.19	44.82	42.74	42.79	45.24	43.86	40.82
Jiangxi	71.81	71.63	71.99	72.02	70.83	70.80	56.02
Shandong	35.45	33.06	30.78	30.15	28.12	27.67	24.74
Henan	66.50	62.74	60.13	58.03	55.16	51.80	43.27
Hubei	79.58	78.14	74.70	68.33	63.49	61.88	52.86
Hunan	85.88	83.00	75.50	76.07	78.75	82.07	75.94
Guangdong	76.57	74.12	71.82	74.66	64.81	58.40	50.40
Guangxi	100.65	96.41	86.27	87.86	82.82	81.12	70.39
Hainan	97.02	97.92	84.18	86.30	85.05	90.22	83.38
Chongqing	76.62	75.00	73.34	77.15	73.37	72.21	66.69
Sichuan	69.68	67.13	65.66	63.12	57.34	60.35	55.19
Guizhou	129.39	133.46	130.66	122.15	114.06	102.51	96.54
Yunnan	56.01	54.42	55.47	57.53	59.61	61.78	60.07
Xizang	147.99	140.20	154.37	154.77	166.66	182.38	150.13
Shaanxi	57.39	56.66	56.30	59.00	55.90	55.55	47.26
Gansu	64.45	54.92	58.13	56.54	38.71	36.18	30.67
Qinghai	101.16	123.26	128.70	134.20	140.33	134.53	93.85
Ningxia	50.84	42.23	40.04	43.00	36.73	36.86	29.74
Xinjiang	176.00	184.53	185.66	202.59	304.94	169.05	109.89

In addition to spatial disparities, the incidence of pulmonary tuberculosis exhibited distinct age-specific distribution patterns. From 2014 to 2020, the incidence rate in each age group increased progressively with age, peaking in the 70-year-old group (329.43/100,000 in 2014), generally following a pattern of “lower incidence in younger age groups and higher incidence in older age groups” (Table 2). Regarding temporal trends, the trajectories of change differed substantially across age groups. On the one hand, most age groups showed a downward trend, with the most pronounced declines observed among the older adults: the 60-year-old group decreased from 275.71/100,000 to 158.30/100,000 (a decrease of 42.6%), the 70-year-old group decreased from 329.43/100,000 to 220.11/100,000 (a decrease of 33.2%), and the 0-year-old group declined continuously from 14.98/100,000 to 10.78/100,000 (a decrease of 28.0%). On the other hand, the 10-year-old and 20-year-old groups showed only minor fluctuations without a clear monotonic trend. These results indicate that although the overall burden of tuberculosis decreased over time, the older adults population—particularly the 70-year-old group—still faces a disproportionately high incidence risk, highlighting the need for precise screening and intervention strategies targeting older adults.

**Table 2:** Incidence rates of pulmonary tuberculosis in different age groups in China from 2014 to 2020 (/100,000).

Age group	2014	2015	2016	2017	2018	2019	2020
0-	14.98	15.35	13.04	12.71	12.35	12.51	10.78
10-	64.95	61.61	62.95	64.04	65.15	71.43	62.48
20-	133.54	125.84	121.86	116.20	108.83	123.31	114.08
30-	108.75	100.15	96.85	96.71	97.13	85.76	74.81
40-	116.56	109.05	103.00	98.68	93.08	92.64	78.07
50-	175.90	173.06	170.77	170.02	171.20	138.95	114.05
60-	275.71	281.69	262.64	269.31	262.57	192.37	158.30
70-	329.43	325.51	277.94	279.66	264.18	268.86	220.11
80-	243.34	258.29	220.10	233.81	222.26	237.04	192.38

### 3.2 Characteristics of the rate of change of each air pollutant relative to the baseline

To further visualize the dynamic evolution of air quality during the study period, 2014 was set as the baseline (value = 0), and the annual relative changes in various air pollutants (PM₂.₅, PM₁₀, SO₂, NO₂, CO, and O₃) from 2015 to 2020 were calculated (Figure 2). The results revealed that the trends of the six pollutants fell into two distinct categories. The first category comprised pollutants with a continuous decline, including PM₂.₅, SO₂, NO₂, and CO; their relative changes remained negative throughout the study period, indicating that concentrations were lower than the 2014 baseline. The second category comprised pollutants with fluctuating increases, including PM₁₀ and O₃, whose relative changes were generally positive.

**Figure 2:**
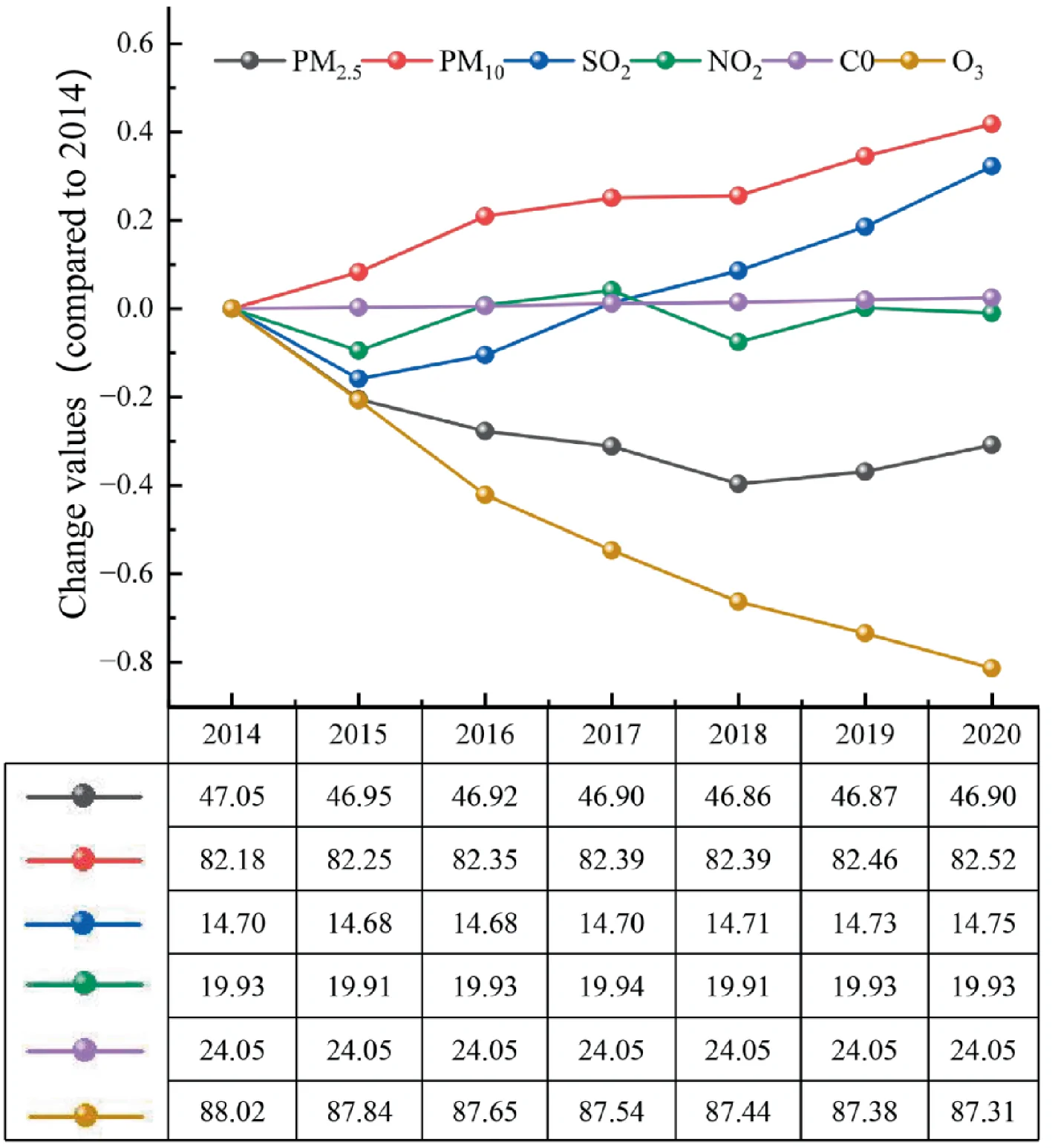
Changing trends in air pollution from 2014 to 2020.

### 3.3 Global spatial autocorrelation analysis

From 2014 to 2020, the Moran I coefficients for each year were all greater than 0, with Z-values exceeding 1.96 and *p*-values less than 0.05. These results indicate positive spatial autocorrelation in pulmonary TB incidence across China, suggesting a clustering pattern. The annual average national incidence from 2014 to 2020 exhibited significant clustering (Table 3). High-high cluster regions included Xinjiang and Xizang. No high-low or low-high clusters were detected. Low-low clusters comprised 12 provincial-level units, including Inner Mongolia, Heilongjiang, Beijing, and Tianjin (Figure 3).

**Table 3:** Global spatial autocorrelation analysis of reported pulmonary TB incidence, 2014–2020.

Year	Moran’s I	E(I)	Variance	Z值	*P*值
2014	0.62	−0.03	0.007	7.70	<0.001
2015	0.58	−0.03	0.007	7.24	<0.001
2016	0.31	−0.03	0.005	4.68	<0.001
2017	0.64	−0.03	0.007	8.18	<0.001
2018	0.54	−0.03	0.005	8.28	<0.001
2019	0.29	−0.03	0.005	4.36	<0.001
2020	0.34	−0.03	0.005	4.97	<0.001
2014–2020	0.66	−0.03	0.007	8.39	<0.001

**Figure 3:**
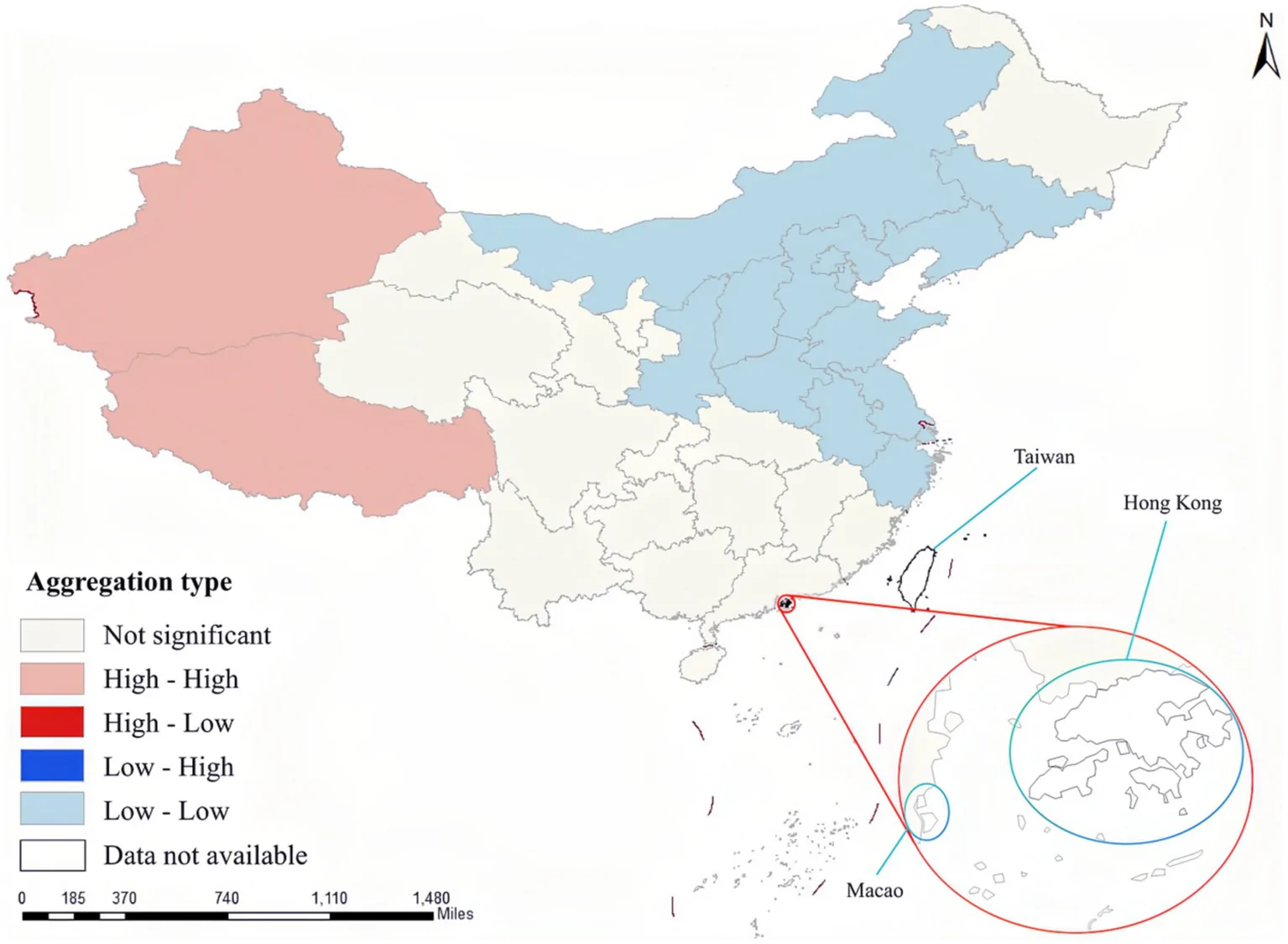
Local autocorrelation analysis of reported incidence rate of pulmonary tuberculosis from 2014 to 2020. The map is sourced from the National Platform for Common Geospatial Information Services, and the review number is GS (2024) 0650. Due to the absence of reported incidence data, Hong Kong, Macao, and Taiwan were not included in the spatial analysis.

### 3.4 Time and space scanning analysis

Spatiotemporal scanning analysis identified a total of 17 statistically significant clusters of pulmonary TB nationwide. Based on their geographic distribution characteristics and epidemiological attributes, these clusters could be classified into two major patterns. The first cluster type was primarily concentrated in three western provinces - Xinjiang, Qinghai, and Xizang - exhibiting a remarkable regional high-incidence pattern. The second cluster type was widely distributed across southwestern and central-eastern regions, covering 15 provinces including Guangxi, Hainan, Guizhou, Guangdong, Yunnan, Hunan, Chongqing, Sichuan, Jiangxi, Hubei, Henan, and Anhui (see Table 4 for details).

**Table 4:** Spatiotemporal clustering analysis of pulmonary TB in China, 2014–2020.

Aggregation level	Aggregation area	Time	Number of people (in 10,000)	Actual cases	Expected cases	RR	LLR	*P*
I	Xinjiang, Qinghai, Xizang	2016–2018	3,327	206,617	59,672	3.56	111606.75	< 0.001
		2014–2014	3,327	50,314	16,216	3.12	22973.85	< 0.001
II-1	Guangxi, Hainan, Guizhou, Guangdong, Yunnan, Hunan, Chongqing, Sichuan, Jiangxi, Hubei	2014–2016	54,841	1,245,295	946,689	1.4	52393.88	< 0.001
II-2	Guangxi, Hainan, Chongqing, Sichuan, Guizhou, Yunnan, Hunan	2018–2019	32,378	487,109	380,668	1.31	14734.00	< 0.001
II-3	Heilongjiang	2014–2016	3,393	98,095	61,807	1.60	9141.11	< 0.001
		2018–2019	3,393	47,187	38,363	1.23	951.67	< 0.001
II-4	Henan	2014–2015	9,809	121,795	112,852	1.08	352.43	< 0.001
II-5	Anhui	2014–2014	6,053	37,290	34,975	1.07	75.44	< 0.001

RR: relative risk; LLR: log-likelihood ratio.

### 3.5 The Association of air Pollution on the incidence of tuberculosis

A Pearson correlation coefficient matrix was first computed to examine pairwise linear associations among all eight candidate independent variables (Table 5). Two pairs exhibited extremely high correlations: PM₂.₅ and PM₁₀ (r = 0.910, *p* < 0.01) and GDP and PVOPBAT (r = 0.872, *p* < 0.01), indicating substantial redundancy. Among the remaining variable pairs, all absolute correlation coefficients were below 0.50, with the highest being that between PM₂.₅ and GDP (r = 0.423, *p* < 0.01).

**Table 5:** Correlation Matrix of Air Pollutants.

Variable	PM_2.5_	PM_10_	SO_2_	NO_2_	CO	O_3_	PVOPBAT
PM_10_	0.910**						
SO_2_	0.344**	0.354**					
NO_2_	0.355**	0.345**	−0.295**				
CO	0.238**	0.313**	−0.160*	−0.353**			
O_3_	0.026	0.048	−0.053	−0.013	0.128		
PVOPBAT	0.423**	0.246**	0.13	0.172*	0.032	0.072	
GDP	0.255**	0.09	−0.033	0.052	0.069	0.256**	0.872**

** Significantly correlated at the 0.01 level (two-tailed); * Significantly correlated at the 0.05 level (two-tailed).

Since the correlation test indicated that PM2.5 and PM10, as well as GDP and PVOPBAT, were highly correlated, multicollinearity was assessed by calculating variance inflation factors (VIF). The VIF values ranged from 1.253 to 9.758 (Table 6). Among them, PM10 (VIF = 9.758, Tolerance = 0.102) and PVOPBAT (VIF = 6.986, Tolerance = 0.143) had the highest VIF values. These results suggested that excluding PM10 and PVOPBAT would not result in substantial loss of information, as the information they carried largely reflected the same variation as that captured by PM2.5 and GDP, respectively. After excluding these two variables, all VIF values decreased to below 3.250 (Tolerance > 0.300), confirming that multicollinearity was effectively controlled and that the final model specification was reasonable.

**Table 6:** Multicollinearity test results.

Before exclusion			After exclusion		
Variable	Tolerance	VIF	Variable	Tolerance	VIF
PM_2.5_	0.128	7.821	PM25	0.308	3.249
PM_10_	0.102	9.758	SO2	0.379	2.641
SO_2_	0.262	3.822	NO2	0.314	3.186
NO_2_	0.219	4.570	CO	0.407	2.459
CO	0.286	3.499	O3	0.912	1.097
O_3_	0.796	1.256	GDP	0.817	1.224
PVOPBAT	0.143	6.986	—	—	—
GDP	0.153	6.526	—	—	—

The GTWR model was constructed, and the results showed that *R*^2^ = 0.889, adjusted *R*^2^ = 0.886, and AICc = 1873.18. The average regression coefficients of the GTWR model are presented in Table 7. The associations between air pollutants, socioeconomic factors, and tuberculosis incidence across 31 Chinese provinces exhibited significant spatial variations (Table 7). Overall, O3 showed consistently negative local coefficients in most regions, with particularly strong inverse associations observed in northeastern provinces (e.g., −0.984 in Heilongjiang, −0.975 in Jilin, and −0.956 in Liaoning), while only Yunnan showed a marginally positive value (0.092). In contrast, PM2.5 displayed a distinct east-to-west gradient: coefficients were slightly negative or near zero in eastern and northern areas (e.g., −0.097 in Beijing, −0.074 in Tianjin, and −0.021 in Hebei) but increased substantially across southwestern and northwestern provinces (e.g., 0.789 in Sichuan, 1.329 in Qinghai).

**Table 7:** Mean regression coefficients of air pollutants on pulmonary TB incidence.

Province	PM_2.5_	SO_2_	NO_2_	CO	O_3_	GDP
Beijing	−0.097	0.099	0.158	0.028	−0.776	3.885
Tianjin	−0.074	0.099	0.202	0.047	−0.852	4.532
Hebei	−0.021	−0.069	−0.010	−0.127	−0.745	0.373
Shanxi	0.049	−0.213	−0.164	−0.266	−0.720	−3.344
Inner Mongolia	−0.020	−0.064	−0.074	−0.150	−0.702	−3.665
Liaoning	−0.227	0.386	0.445	0.292	−0.956	4.906
Jilin	−0.265	0.486	0.500	0.359	−0.975	3.156
Heilongjiang	−0.273	0.543	0.522	0.394	−0.984	1.630
Shanghai	0.064	−0.252	0.099	−0.155	−0.965	1.964
Jiangsu	0.119	−0.318	−0.022	−0.246	−0.948	1.018
Zhejiang	0.122	−0.343	0.022	−0.248	−0.953	0.242
Anhui	0.158	−0.386	−0.096	−0.326	−0.924	−0.843
Fujian	0.211	−0.480	0.006	−0.367	−0.949	−3.567
Jiangxi	0.236	−0.537	−0.166	−0.469	−0.881	−5.186
Shandong	0.030	−0.102	0.070	−0.097	−0.924	3.431
Henan	0.127	−0.359	−0.217	−0.371	−0.789	−3.585
Hubei	0.224	−0.529	−0.245	−0.493	−0.827	−5.671
Hunan	0.263	−0.626	−0.273	−0.574	−0.752	−8.483
Guangdong	0.178	−0.597	0.076	−0.420	−0.809	−7.418
Guangxi	0.087	−0.651	0.160	−0.452	−0.381	−11.239
Hainan	−0.077	−0.558	0.518	−0.239	−0.559	−8.801
Chongqing	0.530	−1.020	−0.817	−1.008	−0.358	−17.149
Sichuan	0.789	−1.285	−1.163	−1.285	−0.315	−21.072
Guizhou	0.393	−0.889	−0.492	−0.841	−0.272	−15.590
Yunnan	0.561	−1.086	−0.555	−1.025	0.092	−19.307
Xizang	1.281	−1.053	−0.254	−1.773	−1.570	−32.929
Shaanxi	0.370	−0.771	−0.654	−0.746	−0.660	−12.219
Gansu	0.990	−1.465	−1.321	−1.344	−0.842	−22.481
Qinghai	1.329	−1.739	−1.548	−1.699	−1.022	−27.360
Ningxia	0.571	−0.964	−0.839	−0.849	−0.926	−15.724
Xinjiang	−3.085	3.200	3.352	0.649	−4.650	−2.793

SO₂, NO₂, and CO exhibited relatively coherent spatial distribution patterns. In northeastern China, all three pollutants showed positive coefficients with tuberculosis incidence (e.g., 0.543, 0.522, and 0.394, respectively, in Heilongjiang). In contrast, pronounced negative coefficients dominated the southwestern and northwestern regions (e.g., −1.739 for SO₂, −1.548 for NO₂, and −1.699 for CO in Qinghai), indicating marked geographical disparities in the direction and magnitude of these associations. Notably, Xinjiang presented extreme outlier values (PM2.5: −3.085; SO₂: 3.200; NO₂: 3.352; O₃: −4.650).

After adjustment for GDP, the local coefficients further revealed a pronounced spatial asymmetry. Eastern provinces generally exhibited positive values (e.g., 4.532 in Tianjin, 3.885 in Beijing, and 4.906 in Liaoning), whereas western provinces showed strongly negative coefficients (e.g., −32.929 in Xizang, −27.360 in Qinghai, and −22.481 in Gansu). This east-to-west reversal points to complex interactions between economic development and environmental health burdens that differ across geographic contexts.

### 3.6 Exposure window analysis

Exposure-window analysis using moving average specifications. To assess whether the observed spatial patterns were reproducible across alternative exposure time scales, we re-ran the GTWR model under four moving-average (MA) exposure windows: MA1 (current-year), MA2 (current and previous 1 year), MA3 (current and previous 2 years), and MA5 (current and previous 4 years), corresponding to 1- to 5-year cumulative exposure windows (Table 8).

**Table 8:** Average GTWR regression coefficients by year and moving-average exposure window, 2014–2020.

year	PM_2.5_	SO_2_	NO_2_	CO	O_3_	GDP
Moving average window	MA1	MA2	MA3	MA4	MA5	MA6
2014	0.003	−0.352	0.009	−0.409	−0.428	−7.357
2015	0.042	−0.366	−0.071	−0.451	−0.452	−6.487
2016	0.055	−0.390	−0.136	−0.483	−0.556	−6.089
2017	0.096	−0.401	−0.107	−0.451	−0.775	−6.526
2018	0.181	−0.467	−0.036	−0.382	−1.096	−7.245
2019	0.247	−0.519	−0.125	−0.420	−1.488	−7.441
2020	0.406	−0.143	−0.170	−0.556	−1.466	−10.098
Moving average window	MA2	MA3	MA4	MA5	MA6	MA7
2015	0.023	−0.359	−0.032	−0.430	−0.440	−6.915
2016	0.048	−0.378	−0.104	−0.467	−0.505	−6.284
2017	0.075	−0.396	−0.121	−0.467	−0.666	−6.307
2018	0.139	−0.434	−0.071	−0.417	−0.935	−6.885
2019	0.214	−0.493	−0.080	−0.401	−1.292	−7.343
2020	0.327	−0.331	−0.147	−0.488	−1.477	−8.770
Moving average window	MA3	MA4	MA5	MA6	MA7	MA8
2016	0.034	−0.370	−0.068	−0.448	−0.480	−6.630
2017	0.064	−0.386	−0.105	−0.462	−0.596	−6.366
2018	0.111	−0.420	−0.093	−0.439	−0.809	−6.620
2019	0.175	−0.463	−0.089	−0.418	−1.120	−7.071
2020	0.278	−0.376	−0.110	−0.452	−1.350	−8.261
Moving average window	MA5	MA6	MA7	MA8	MA9	MA10
2018	0.076	−0.396	−0.069	−0.435	−0.666	−6.734
2019	0.125	−0.429	−0.095	−0.437	−0.876	−6.759
2020	0.197	−0.384	−0.115	−0.458	−1.076	−7.480

Average PM2.5 coefficients remained non-negative under all four windows (ranging from 0.003 under MA1 to 0.406 under MA5), reflecting that the underlying positive signal in western provinces was not offset entirely by negative coefficients elsewhere and was reproducible across exposure definitions. SO_2_, NO_2_, and CO all yielded consistently negative average coefficients (SO_2_: −0.143 to −0.519; NO_2_: −0.032 to −0.170; CO: −0.382 to −0.556), indicating that the inverse associations observed in the provincial results were robust to exposure smoothing.

A distinct dose–window pattern emerged for O_3_: coefficients were most strongly negative under the shortest window (MA1: −1.488 in 2019) and attenuated progressively as the window lengthened (MA5: −0.878 in 2019), suggesting that the estimated O_3_–TB association is driven primarily by short-term rather than cumulative long-term exposure. GDP coefficients remained strongly negative across all windows (−6.089 to −10.098), indicating that the socioeconomic gradient in TB incidence is reproducible regardless of exposure time scale.

Integration of provincial and exposure-window findings. The provincial GTWR results (Table 7) and the MA-averaged coefficients (Table 8) together demonstrate that the pollutant–TB relationship in China is both spatially non-stationary and temporally consistent. The spatial divergence observed at the provincial level—positive in the high-burden western regions and negative or near-zero in the eastern regions—explains why nationally aggregated averages remained close to zero for PM2.5 despite consistent directional signals within subregions. Crucially, the stability of the average coefficients across all four MA windows indicates that these spatial patterns are not artifacts of annual fluctuations but reflect persistent geographic structures. Conversely, the attenuation of O₃ effects under longer MA windows reveals that exposure duration itself modifies the estimated association, highlighting that some pollutant effects are acute in nature, whereas others persist or accumulate over time.

## 4 Discussion

### 4.1 Spatiotemporal distribution and overall heterogeneity

The onset of pulmonary tuberculosis is influenced not only by the characteristics of the pathogen but also by transmission conditions, immune status of the host, and the level of economic and social development (22). Previous systematic reviews have indicated significant heterogeneity in the association between air pollutants and tuberculosis across different studies (9, 10). However, much of this evidence is derived from single-center city or national average effect estimates, which fail to capture the substantial regional differences among various provinces in China. Utilizing panel data from 31 provincial administrative regions spanning 2014 to 2020, this study assessed the spatio-temporal non-stationary effects of six air pollutants and GDP on the incidence of pulmonary tuberculosis through the geospatio-temporal weighted regression (GTWR) model. The model demonstrated a strong goodness of fit (*R*^2^ = 0.889). The overall incidence rate reported across the country has decreased. However, Xinjiang and Xizang continue to be high-incidence aggregation areas (RR = 3.56). The PM_2.5_ coefficient exhibits a spatial gradient characterized by “negative in the east and northeast, positive in the southwest and northwest.”. In the northeastern provinces, SO_2_, NO_2_, and CO consistently exert a positive effect, whereas O_3_ demonstrates a negative correlation in most provinces nationwide. The GDP coefficient exhibits a reversal, being “positive in the east and negative in the west”. A single national regression coefficient fails to capture this significant regional heterogeneity. Therefore, regionally differentiated prevention and control strategies are more effectively targeted in practice.

### 4.2 PM_2.5_ effect: east–west gradient and regional behavioral modification

The positive coefficient observed in the west is consistent with the positive association reported in national multicenter studies, whereas the negative coefficient in the east suggests marked regional heterogeneity in this association. Spatiotemporal models from these multicenter studies demonstrated a positive correlation between PM_2.5_ and its primary components with the risk of pulmonary tuberculosis (23). Wang et al. conducted a time series analysis across 22 cities and identified a J-shaped curve linking PM2.5 levels to tuberculosis incidence. The risk of developing tuberculosis was 26% higher among individuals exposed to the highest concentrations than among those at the lowest risk point. Approximately 20% of tuberculosis cases were attributable to PM_2.5_ exposure (24). Additionally, geodetector analysis indicated that PM_2.5_ is the principal factor influencing the spatial distribution of pulmonary tuberculosis, with a Q value of 0.31 (8). A negative coefficient of −0.097 was observed in the eastern regions Beijing. In contrast, a coefficient of 1.281 was recorded in Xizang. Because GTWR fits a linear relationship within each local unit, when PM_2.5_ concentrations in a province fall within the low-concentration range, the local linear approximation may yield near-zero or even negative coefficients—a disparity that may reflect not only different exposure levels but also the high level of economic development, superior medical resources, and low background incidence rates in the eastern regions, all of which complicate the manifestation of the net effects of pollutants. Regional behavioral differences are significant and warrant attention. In the Northeast and the high-cold regions of the north, winters are prolonged. Residents typically adopt protective measures, such as wearing masks and limiting outdoor activities, which effectively reduces the actual inhalation dose of PM_2.5_ and may partially mitigate the immune damage caused by pollutants. Conversely, in certain areas of the southwest and northwest, winters are relatively mild, leading to fewer protective actions. Consequently, high concentrations of pollutants in the external environment are more likely to translate into actual internal exposure. The negative correlation coefficient between PM2.5 levels in Xinjiang and Hainan contrasts sharply with that of other western provinces. All of these areas are marginal regions, and local collinearity may be magnified, leading to unstable coefficient estimations and even extreme values (25). These methodological considerations mainly affect the interpretation of local coefficients and do not weaken the main findings of this study - namely, the significant spatial–temporal heterogeneity and overall positive correlation between air pollution and tuberculosis incidence. The moving-average exposure window analysis makes the research results tend to be normal. From the perspective of exposure sources, the vast desert landscape of this region causes particulate matter (PM) to mainly consist of natural mineral dust, whose physical and chemical properties are significantly different from PM2.5 produced by coal burning and vehicle emissions in the eastern region. However, the health policy implemented in 2018 and the subsequent 2019 epidemic may have contributed to fluctuations in tuberculosis incidence, representing potential contextual factors that should be considered when interpreting the local PM2.5 coefficients.

### 4.3 Synergistic positive effects of SO_2_, NO_2_, and CO in Northeast China

The consistent positive effects of SO_2_, NO_2_, and CO in the three northeastern provinces, with SO_2_ coefficients ranging from 0.386 to 0.543, align with the findings of the multi-city time series study. Research conducted in Chengdu indicated that the risk of active tuberculosis increased when NO_2_, and SO_2_ levels exceeded certain thresholds (12). A study in Urumqi reported a rise in incidence linked to SO_2_ and NO₂ 3 months post-exposure (26). Analyses across multiple provinces and cities estimated that for every 10 μg/m^3^ increase in SO_2_ and NO_2_, the risk of tuberculosis rose by 1.97 and 1.30%, respectively (13). Systematic reviews indicated that for every 1 ppb increase in SO_2_, the risk increased by 1.6%, while for every 1 ppb increase in NO_2_, the risk rose by 1.0% (9). Another review confirmed the association with SO_2_, but NO_2_ did not achieve statistical significance (10). The winter coal-burning heating in Northeast China has increased pollutant concentrations. Data from Urumqi indicate that pollutant levels are closely associated with the heating period (26). National monitoring data reveal that tuberculosis reports peak in January and March each year (27). This temporal overlap suggests that exposure to pollutants may hasten the onset of the disease by impairing local lung immunity and facilitating the reactivation of latent infections.

### 4.4 Negative association of O_3_: photochemical confounding and ecological Bias

O_**3**_ exhibits a negative correlation in the majority of provinces nationwide, with the negative coefficient in Heilongjiang reaching −0.984. This finding contrasts with the results of a 16-city study in Anhui Province, which indicated that O_3_ exposure increases the risk of tuberculosis outpatient visits (28). However, it aligns with the positive effect of O_3_ deficiency observed in the township-level analysis in Ningxia (29) and the negative correlation of O_3_ identified in the endogenous reactivation study utilizing whole genome sequencing (30). Theoretically, the strong oxidizing properties of O_3_ may impair the survival of mycobacteria in the air. Additionally, its peak concentration season typically coincides with intense ultraviolet radiation and optimal ventilation. These photochemical conditions, along with alterations in the tuberculosis transmission environment, may lead to temporal confusion in the observed relationships. The photochemical reactions of O_3_ with particulate matter (PM) and nitrogen dioxide (NO₂) may contribute to the negative coefficient observed in the single-pollutant model, which could be confounded by residual bias that inadequately accounts for co-pollutants. This negative correlation is interpreted as a lack of mechanistic support for the proposed protective effect.

### 4.5 Non-pollutant drivers: socioeconomic factors and high-burden clusters

The relationship between economic activities and the burden of disease exhibits spatial non-stationarity. The inverse pattern of “positive in the east and negative in the west” regarding the GDP coefficient suggests that socio-economic factors and the burden of tuberculosis are not merely linear. Nationwide evidence indicates that internal migration significantly influences the spatial spread of tuberculosis: clusters of sputum smear-positive pulmonary tuberculosis spatially overlap with clusters of population outflow, and cases in outflow areas may association the epidemiological characteristics of adjacent provinces (31). In the economically active eastern regions, there is a frequent influx of population and a concentration of transportation hubs. The opportunities for exposure, along with delayed diagnosis and treatment resulting from the migration process, may elevate the risk of transmission. The adverse association of GDP in the western region indicates that economic growth has not yet automatically translated into health benefits. Global ecological analyses demonstrate that a higher proportion of social protection expenditure relative to GDP correlates with a lower risk of tuberculosis infection, onset, and mortality (32). Additionally, national longitudinal studies have identified the Human Development Index and social protection as significant determinants of disease trends (33). The GWR study conducted in Xinjiang revealed that the direction and intensity of the same socio-economic factor varied across different regions, indicating non-stationarity (34). This finding supports the GTWR results of the current study. Total GDP alone does not adequately account for regional disparities; instead, the level of social protection and the efficiency of public health expenditure conversion are more critical factors.

### 4.6 Exposure-window analysis of pollutant–TB associations: temporal robustness and dose–window patterns

The exposure-window sensitivity analysis provided critical insights into the temporal robustness of the observed spatial heterogeneity. The fact that average PM₂.₅ coefficients remained non-negative across all moving-average windows (ranging from 0.003 under MA1 to 0.406 under MA5) suggests that the positive signal in western provinces is not an artifact of annual fluctuations but a persistent geographic structure. This finding aligns with previous multi-city time-series studies that reported cumulative PM₂.₅ effects on tuberculosis risk (35, 36), and extends them by demonstrating that the spatial divergence—positive in high-burden western regions, negative or near-zero in eastern regions—cannot be explained away by exposure smoothing. Had the positive western signal been driven by short-term anomalies, it would have attenuated under longer windows; the opposite pattern (strengthening from MA1 to MA5) instead implies that cumulative long-term exposure amplifies regional disparities.

The consistent inverse associations for SO₂, NO₂, and CO across all windows (−0.143 to −0.519, −0.032 to −0.170, and −0.382 to −0.556, respectively) further corroborate the provincial GTWR findings. These results are consistent with the synergistic positive effects of winter coal burning in northeastern China reported in the literature (9–12), but the negative averages at the national level highlight that region-specific drivers dominate the net association. This underscores a key message: national pooled estimates of air pollution–TB associations are not only statistically unstable but also epidemiologically misleading, as they mask opposing directions of effect in different regions.

A particularly noteworthy dose–window pattern emerged for O₃: the strongest negative coefficient was observed under the shortest window (MA1: −1.488 in 2019), with progressive attenuation as the window lengthened (MA5: −0.878 in 2019). This indicates that the estimated O₃–TB relationship is driven primarily by short-term exposure, which is consistent with the photochemical and seasonal confounding mechanisms discussed earlier (Section 3.4). Unlike PM₂.₅, whose effects accumulated over time, O₃ appears to operate through acute rather than chronic pathways—a distinction that has direct implications for prevention: short-term air quality warnings may be more relevant for O₃-related TB risk than long-term emission reduction targets, whereas the opposite holds for PM₂.₅ in western China.

Finally, the persistently negative GDP coefficients across all windows (−6.089 to −10.098) reinforce that the socioeconomic gradient in TB burden is temporally invariant. This finding resonates with global ecological evidence that social protection expenditure relative to GDP—not GDP alone—drives tuberculosis trends (9, 22).

In summary, the exposure-window analysis demonstrates that the spatial non-stationarity identified by GTWR is not a statistical artifact of annual variability, but a robust, temporally consistent feature of pollutant–TB relationships in China. Future studies should therefore move beyond single-year or national-average designs and adopt spatiotemporally resolved frameworks capable of capturing both geographic and temporal modifiers of environmental health risks.

### 4.7 Limitations

This study is an ecological study. The data consist of provincial aggregated figures and do not account for variations in individual exposure, lifestyle, immune status, or other confounding factors, which may lead to ecological fallacies. In addition, the reported incidence rate is affected by the underreporting rate of the web-based direct reporting system, which may result in an underestimation of the true disease burden. Furthermore, introducing interaction terms into the GWR-type models adopted in this study could substantially exacerbate local collinearity, thereby precluding detection of the interactive effects among different pollutants.

## 5 Conclusion

Pulmonary TB incidence declined overall in China from 2014 to 2020, but western provinces—especially Xinjiang, Xizang, and Qinghai—remained the highest-burden areas, with pronounced spatial clustering. Air pollutant–TB associations varied markedly across provinces, supporting region-specific prevention strategies prioritizing these high-risk areas. As an ecological study, this investigation identifies associations but not causal relationships.

## Data Availability

The original contributions presented in the study are included in the article/supplementary material, further inquiries can be directed to the corresponding author/s.

## Glossary

CI: Confidence interval
CO: Carbon monoxide
COVID-19: Coronavirus disease 2019
DR-TB: Drug-resistant tuberculosis
DOTS: Directly observed treatment short-course
GDP: Gross domestic product
GTWR: Geographically and temporally weighted regression
LLR: Log-likelihood ratio
LISA: Local Indicators of Spatial Association
Moran’s I: Moran’s Index (spatial autocorrelation index)
MTB: *Mycobacterium tuberculosis*
ndp: National TB Control Program
NO_2_: Nitrogen dioxide
O_3_: Ozone
PM_2.5_: Particulate matter ≤2.5 μm
PM_10_: Particulate matter ≤10 μm
PVOPBAT: Passenger volume of public buses and trolleybuses
RR: Relative risk
SO_2_: Sulfur dioxide
TB: Pulmonary tuberculosis/Tuberculosis
VIF: Variance inflation factor
WHO: World Health Organization
